# Nano-Biomaterials for the Delivery of Therapeutic and Monitoring Cues for Aortic Diseases

**DOI:** 10.3389/fbioe.2020.583879

**Published:** 2020-11-05

**Authors:** Shichao Zhu, Kai Zhu, Jun Li, Hao Lai, Chunsheng Wang

**Affiliations:** ^1^Department of Cardiac Surgery, Zhongshan Hospital, Fudan University, Shanghai, China; ^2^Shanghai Institute of Cardiovascular Diseases, Shanghai, China

**Keywords:** nano-biomaterials, aortic diseases, therapeutics, drug delivery, regeneration

## Abstract

The aorta is the largest artery in the body, so any diseases or conditions which could cause damage to the aorta would put patients at considerable and life-threatening risk. In the management of aortic diseases, the major treatments include drug therapy, endovascular treatment, and surgical treatment, which are of great danger or with a poor prognosis. The delivery of nano-biomaterials provides a potential development trend and an emerging field where we could monitor patients’ conditions and responses to the nanotherapeutics. One of the putative applications of nanotechnology is ultrasensitive monitoring of cardiovascular markers by detecting and identifying aneurysms. Moreover, the use of nanosystems for targeted drug delivery can minimize the systemic side effects and enhance drug positioning and efficacy compared to conventional drug therapies. This review shows some examples of utilizing nano-biomaterials in *in vitro* organ and cell culture experiments and explains some developing technologies in delivering and monitoring regenerative therapeutics.

## Introduction

Aortic diseases are generally defined as conditions that affect any part of the aorta, including the chest (thoracic aorta) and abdomen (abdominal aorta). It always involves a focal dilation of the vessel due to structural aortic diseases (aneurysm, dissection, or rupture), atherosclerosis, or other connective tissue disorders (Marfan Syndrome, Ehlers-Danlos syndrome), which can be fatal if not being treated. Due to the different pathological mechanisms, this review just focused on structural aortic diseases. According to the epidemiology of aortic aneurysm in the United States, 104,458 people died from 1999 to 2016 (Abdulameer et al., 2019), which is one of the leading causes of death. Current mainstream therapy for aortic aneurysms concerns invasive treatments, including open-chest surgery to replace the damaged section with vascular prostheses and endovascular implantation of stent-grafts; however, clinically few drugs could be applied to inhibit the occurrence of aortic aneurysms (Hiratzka et al., 2010). Meanwhile, the inherent differences in imaging modalities and measurements performed by different providers, both in terms of method and spatial resolution, may severely affect the ability of the primary based imaging diagnosis even leads to morbidity and mortality. Therefore, there is a need for the development of improved treatments and imaging techniques for aortic diseases.

Nano-biomaterials, as the technology of materials on an atomic, molecular, and supramolecular scale, have shown promises for clinical application. Over recent decades, a wide variety of nanomaterials has been designed and widely tested in preventing, diagnosing, and curing disease or repairing damaged tissues in animal models. More recently, by improving the development of nano-scale technologies and our understanding of conditions at the molecular level, nano-biomaterial has witnessed a growth explosion in the world and offers extraordinary openings for overcoming the restrictions of conventional biomaterials. It motivates several novel therapeutic approaches to the modernization of the treatment of aortic diseases. Nanoparticles (NPs), the particles in nanometric range (1–100 nm) and over 100 times smaller than human cells (Suri et al., 2007; Wilczewska et al., 2012; Au et al., 2016), are bioactive and mobile in both intra- and extravascular systems (Buzea et al., 2007). They have shown significant potential to provide a platform for targeted delivery of drugs and imaging agents because of their unique multi-functionality, such as the high penetration, prolonged blood half-life, and image contrasting capacity; they can also avoid removal by the reticuloendothelial system (Zhao et al., 2014; Au et al., 2016). In addition, nanomaterials provide a suitable platform for functionalization with other moieties, so that multiple functions can be combined onto NPs. Different types of targeting ligands, like peptides and inflammation cues, can be conjugated to NPs for targeting the morbid sites (Jiang et al., 2017). On the other hand, NPs can be conjugated with functional ligands to evade rapid phagocytic clearance from blood circulation (Rodriguez et al., 2013; Jiang et al., 2017). A novel approach for tracking, sensing, and triggering *in vivo* therapeutics using nano-biomaterials and soft bio-electronics for the subsequent development of minimally invasive and regenerative therapies (MIRET) offers tremendous opportunities (Ashammakhi et al., 2019).

This review highlights the recent advances in nano-biomaterial application in aortic disease therapy. The technologies in delivering and monitoring regenerative therapeutics based on nanotechnical approaches are also discussed.

## Aorta Diseases and Animal Models

Aortic aneurysm is a leading cause of mortality and morbidity among the elderly (Golledge, 2019), and accounts for >25000 deaths in the United States annually (Quintana and Taylor, 2019). Pathologically, extracellular matrix (ECM) proteins broken down by matrix metalloproteinases (MMPs), reactive oxygen species (ROS) overproduction, inflammatory cell infiltration of vessel walls, depletion of vascular smooth muscle cells (VSMCs), and elasticity lamellar aortic degeneration have been widely proven to be responsible for aneurysm formation (Joviliano et al., 2017; Quintana and Taylor, 2019). Furthermore, biomechanical forces work in concert with these structural and inflammatory processes to cause aneurysm dilation, rupture, and dissection (Hiratzka et al., 2010; Nordon et al., 2011). Given the complex pathology process of aortic diseases, it is essential to develop and select a suitable and stable animal model for the investigation of nano-biomaterials. Several aortic disease animal models, including surgical induction and chemical induction, have been developed in the past decades. However, most of them cannot model the real pathogenesis of aortic diseases. Up to date, only few of them had been successfully utilized for the evaluation of *in vivo* behaviors of nano-biomaterials. Among them, induction by calcium chloride (CaCl_2_) and angiotensin II (AngII) were the most widely used animal models for research (Sénémaud et al., 2017).

One of the ordinary and brief animal models involves the utilization of calcium chloride (CaCl_2_) around the infrarenal aorta in rats or mice for abdominal aortic aneurysm (AAA). Briefly, AAA induction was performed by placing a cotton gauze strip soaked with CaCl_2_ directly to the abdominal aorta for 10 min, and AAA formed 3 weeks after surgery (Chiou et al., 2001). Histological examination demonstrated that aortic dilatation was accompanied by VSMC depletion, elastin degradation, and infiltration of lymphocytes and macrophages. High concentrations of pro-inflammatory cytokines and MMPs were also detected within the dilated aortas (Chiou et al., 2001; Longo et al., 2002; Yoshimura et al., 2006; Li et al., 2017). The significant advantage of this model is that it reveals a clear inflammatory infiltrate, including macrophages, calcification of medial elastin, ROS production, and VSMCs apoptosis, comparable to the clinical appearance (Wang et al., 2013).

Angiotensin II-induced rat or mice model is also widely used for its availability in resulting in both thoracic aortic aneurysm (TAA) and AAA. The apolipoprotein E (apoE)^–/–^ mice were systemically infused with Ang II *via* subcutaneous osmotic pumps over a prolonged period, the advantage of which is the presence of atherosclerosis, a risk factor shared with human patients (King et al., 2009; Sénémaud et al., 2017). More critically, aneurysm rupture, which is a clinically well-established complication, could only be seen in this model (Barisione et al., 2006). To explore mechanisms of the complete and fatal process, it was necessary to improve the rate of aortic aneurysm rupture. Therefore, Wang et al. (2010) innovated a model in which C57BL/6 wild-type mice were given both AngII and anti-transforming growth factor (TGF-β) antibodies to promote AAA formation, and the incidence of aneurysm rupture occurred at an increased rate to 80%.

Intriguingly, there was a specific animal model that was not accessible but of great potential. Wang et al. (2019) created an AAA model induced by chronic infusion of AngII into low density-lipoprotein receptor-deficient (LDLr^–/–^) mice, together with a high-fat diet. It is generally accepted that aneurysm formation occurs when the aorta dilates more than 1.5 times its standard outer diameter (Kontopodis et al., 2016). Based on this criterion, the incidence of AAA in this model is 81.82%, which is higher than that in normal AngII-infused mice (56%) (Trachet et al., 2015). The team suggested that this may be due to the prolongation of aneurysm formation time from 28 to 42 days (Cassis et al., 2009). Histological findings demonstrate pathological features such as elastic laminar degeneration and atherosclerosis, which do not differ from the AngII-induced ApoE^–/–^ genetic background mouse model (Lysgaard Poulsen et al., 2016). There are several methods of modeling: surgical injection of elastase, calcium chloride patch, AngII pumping (ApoE^–/–^ or LDLr^–/–^, added other factors such as Anti-TGFb, BAPN, etc.).

## Nano-Biomaterials Used for Delivering and Imaging in Animal Models

The enormous potential of nano-biomaterials has shown a tendency that creates a nano-scale platform for targeted delivery of drugs and imaging agents to its intended site and improved strategies for aortic disease treatment. Nanomedicine is now being used in a variety of diseases, especially in cancers (Au et al., 2016). NP-based biofilms have also been applied for infection screening (Thet et al., 2016).

The use of optimized nanocarriers, such as liposomes, gold, and iron oxide, can successfully deliver NPs to targets in diseased or healthy tissues for therapeutic or diagnostic use (Suri et al., 2007; Jain, 2010; Zhao et al., 2014). To maximize targeting accuracy and minimize off-target effects in aortic disease, researchers should understand the pathology of aortic diseases and choose the most appropriate biomarkers. Although biomarkers like degraded elastic lamina, inflammatory cells, and ROS are available to target vasculature and deeply associated with the development of aorta pathology, which target would be the most stable and accessible one remains to be discussed. For example, infiltration of the vessel wall by macrophages and monocytes contributes to both TAA and AAA progression, but their highly heterogeneous state and being rapidly recycled by receptors made it difficult to be designed as a reliable indicator of the risk of chronic aneurysm rupture (He et al., 2008; Meng et al., 2016). Another one of the most consistent features of aortic disease is elastic layer fragmentation and degradation. The adult elastic layer does not undergo substantial remodeling in the timeline of disease progression or aneurysm wall degradation, making it an ideal target for delivering NPs.

Herein, we summarized the delivery of therapeutic and imaging agents in aortic diseases (Tables 1, 2).

**TABLE 1:** Nanoparticles for therapies.

Nanoparticles	Target	Loaded cargo	Animal model	Application
PLA NPs	Degraded elastin layer	DIR	CaCl_2_-induced rat model	Locating diseased vessels and avoiding normal areas (Sinha et al., 2014)
PLA NPs		BB-94		Suppress MMP activity and development of aorta (Nosoudi et al., 2015)
BSA NPs		EDTA		Remove calcification in aneurysm (Lei et al., 2014)
BSA NPs		EDTA and PGG		Reduced macrophage recruitment, MMP activity, calcification, and elastin degradation (Nosoudi et al., 2016)
BSA NPs		PGG	PPE-induced mice model	Reversed the inflammatory marker (Dhital and Vyavahare, 2020)
Poly (ethylene glycol)-b-poly (γ-benzyl L-glutamate) (PEG-b-PBLG)	Defect (disruption and degeneration) in the aneurysm wall	RAP	elastase-induced rat model	AAA formation and wall inflammation were suppressed (Shirasu et al., 2016)
ROS-responsive NPs	ROS	RAP	CaCl_2_-induced rat model	Reduced aneurysm diameter and removed calcification (Cheng et al., 2018)

**TABLE 2:** Nanoparticles for imaging.

Nanoparticles	Target	Measurements	Animal model	Application
EL-GNP	Degraded elastin	micro-CT	AngII-induced AAA mice	Predict the level of elastin injury and the possibility of rupture (Wang et al., 2019)
FDG-NPs	macrophages	micro-PET/CT		NPs absorption was associated with aorta expansion (Nahrendorf et al., 2011)
USPIO-NPs		MRI	Human patients	Predict expansion rates (Richards et al., 2011)
CNA-35 NPs	type 1 collagen		AngII-induced AAA mice	Predict its risk of rupture (Klink et al., 2011)
Iron oxide NPs	Integrin			detect high-risk atherosclerotic and aneurysmal vascular diseases (Kitagawa et al., 2012)
Iron oxide NPs	MMPs			Predict its risk of rupture (Yao et al., 2020)

### Delivery of Therapeutic Cues in Aortic Diseases

As mentioned before, traditional treatments for the aortic disease possess many risks and side effects. Therefore, it is of great importance to develop a safe and effective drug treatment, which can retard the disease progression and alleviate the need for surgery. Recently, certain drugs targeting the dilation or rupture of aneurysms have been investigated in animal models and have yielded remarkable results on the inhibition of aneurysm formation (Steinmetz et al., 2005; Baxter et al., 2008; Shiraya et al., 2009; Golledge et al., 2010; Habashi et al., 2011). However, these promising drugs still need further research to elevate their efficacy in clinical trials, potentially because oral and parenteral or intra-arterial administrations would result in unwanted adverse effects by other organs and limited doses of drugs sent to the wall due to the rapid blood flow with high shear (Karlsson et al., 2009; Kurosawa et al., 2013). For instance, it is minimally effective for systemic doxycycline administration in AAA and has severe side-effects, including cutaneous photosensitive reactions, tooth discoloration, gastrointestinal symptoms, and yeast infection (Baxter et al., 2002). The emergence of nano-biomaterials solves a series of problems, including high shear blood flow, lack of stable binding sites, heterogeneity, and recycling of cellular markers. Nanoscience provides a possibility that NPs can bind specific areas like degraded elastic lamina, inflammation cells, or peptide. The varied nano-biomaterials can exist stably in the blood vessels for a long time without being taken up by cells. Furthermore, it was surprising that they can control the drug release rate by detecting the extent of vascular damage.

Sinha et al. (2014) used poly(D,L-lactide) (PLA) NPs to load 1,1-dioctadecyl-3,3,3,3-tetramethylindotricarbocyanine iodide (DIR) dyes, and surface maleimide groups conjugated to thiolate elastin/IgG antibodies, targeted for degraded elastin layer. DIR dyes were indelibly associated with particles to facilitate visualization and tracking of NPs. First, they used isolated rat aorta treated with elastase to simulate elastin degradation *in vitro* and found that the NPs and elastase attachment efficiency increased with greater elastic damage. Furthermore, they accessed the targeting efficiency with a prevalent aortic aneurysm model (CaCl_2_-induced rat model) and the other two vascular disease models (atherosclerosis and vascular medial calcification). CaCl_2_ can mimic the degradation of the elastic lamina in the abdominal aorta of rats (Basalyga et al., 2004; Isenburg et al., 2007), and the results strongly suggest that NPs are sensitive to elastic injury and precise spatial accumulation even under high-shear hemodynamic conditions. The next plan is to assay these NPs in animals with biochemical damage rather than local chemical injury (Sinha et al., 2014).

Besides elastic lamina degeneration, vascular calcification is also a common feature in the pathology of aortic diseases. Nosoudi et al. (2015) have designed an elastin-antibody binding PLA NP loaded with hydroxamic acid MMP inhibitor Batimastat (BB-94), which could suppress AAA in systemic delivery. The activity of MMP was completely 56% lower at the injury site compared to thoracic aorta, and the development of aorta diameter was significantly inhibited by NPs (40.25 ± 26%) compared to control (269.5 ± 56%). Based on these, Lei et al. (2014) further innovate bovine serum albumin (BSA) NPs loaded with ethylenediaminetetraacetic acid (EDTA) and deliver them to the aneurysm site to remove calcification (proved by Alizarin red staining and CT).

**FIGURE 1:**
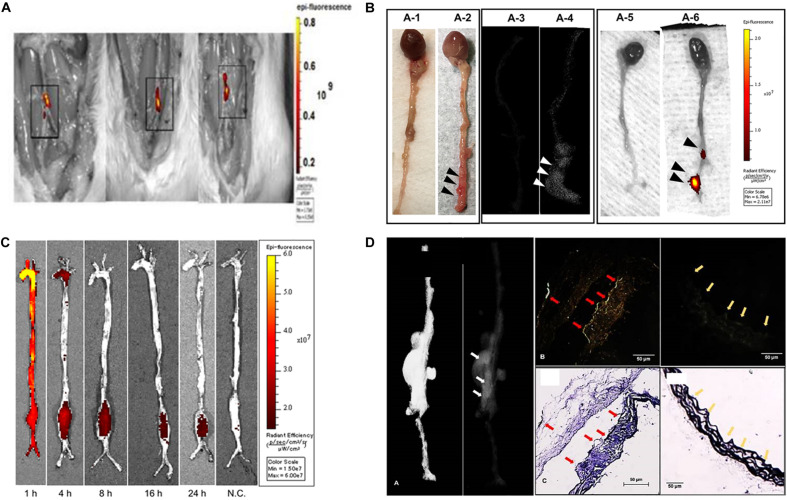
Animal models of aortic aneurysms. **(A)** NP accumulation after intravenous injection of EL-NP-DIR, 30 days after injury at the site of elastin damage. **(B)** Representative picture showing targeting to degraded elastin. **(C)** Accumulation of rapamycin nanoparticles in the AAA rat model. **(D)** Localization of EL-GNPs within aneurysmal tissues. Color arrows and arrowheads refers to the stronger signal was found in the B1 which showed more elastin damage (C1) than the control section (B2) that contained only intact elastin fibers (C2).

Although both approaches have some effects in animal models, the common issue is that they are only valid for the early stages of aortic aneurysms. To simulate the clinical condition and treat moderate-sized aneurysms, Nosoudi et al. (2016) innovate a dual-targeted approach using NPs to remove deposits of minerals and restore the elastic layer to reverse the development of moderate aneurysms in CaCl_2_-induced AAA rat models. EDTA was first administered to eliminate calcified deposits in the arteries; and then to release the polyphenol, pentagalloyl glucose (PGG) stabilizes elastin and enhances elastic fiber deposition (Figure 1A). By delivery of the chelators EDTA and PGG, macrophage recruitment, MMP activity, calcification, and elastin degradation in the aorta were all ignorantly reduced, which led to the improvement in vascular elastance. Dhital and Vyavahare (2020) also designed a PGG-loaded nanoparticle tested in porcine pancreatic elastase (PPE)-induced AAA mice model. They used fluorescence staining to confirm its targeting and increased the circumferential strain of the aneurysm aorta to normal levels by reversing the inflammatory marker (Figure 1B). From these results, dual treatment may be a potential option for regulating aneurysm development.

The previous analysis of the histopathology of aneurysm specimens suggests that abundant structural micro-defects may be to blame for the destruction and degeneration of aortic aneurysms (Campa et al., 1987; Dobrin and Mrkvicka, 1994; Thompson et al., 1995; López-Candales et al., 1997). Therefore, Shirasu et al. (2016) designed a special nanosystem that uses the penetration of NPs to make them reach the defect in the aneurysm wall. They chose elastase-induced AAA rat model to inject the NPs containing rapamycin (RAP, a candidate that has been reported to inhibit aneurysm formation). Microscopic analysis showed that the rapamycin NPs successfully bound to the damaged structures of the wall (Lawrence et al., 2004). Furthermore, AAA formation and wall inflammation were markedly suppressed as compared to RAP treatment alone at 7 days after elastase infusion. Accordingly, MMP activity and expression of inflammatory cytokines were also significantly depressed. All results indicate that the NP-based delivery system enables drug targeting delivery and has great potential in drug therapy targeting aneurysm (Figure 1C).

Based on the methods mentioned before, Cheng et al. (2018) developed a nano-therapy that was responsive to ROS and could release RAP. Meanwhile, based on the pathophysiological process of aortic aneurysm closely associated with ROS, they developed a multifunctional nano-therapy for targeted treatment. They used the nanoplatform composed of ROS-responsive materials to target aneurysmal sites and can release therapeutic molecules when triggered by ROS. With CaCl_2_-induced AAA rat models, reduced aneurysm diameter and removed calcification were examined *in vivo*. Intriguingly, the essential MMPs (MMP-2, MMP-9) involved in aortic diseases were remarkably decreased. The same limitations they faced were therapeutic benefits and safety profiles, which still need to be examined in other animal models.

### Delivery of Imaging Cues in Aortic Diseases

Imaging is vital for patients with aortic diseases, as most patients remain asymptomatic until they develop a severe complication such as dissection or rupture. The only way to get an early diagnosis is to use imaging cues such as ultrasound, CT, and echocardiography for measuring the size of the aneurysm and monitoring its growth rate (Khalil et al., 2007; Baliga et al., 2014). Although imaging has rapidly progressed in recent years, early diagnosis of aortic disease is still an urgent issue to be addressed. Even the decision of whether to perform surgery or endovascular repair requires imaging results of aneurysm diameter and rate of expansion.

As mentioned previously, accurate anatomical and morphological measurements of aortic aneurysms are critical for diagnosis and long-term follow-up, so contrast-enhanced CT is a commonly used imaging modality. However, the disadvantage of this technique is also apparent in that it can only provide morphological information but no specific pathological information. If the degree of ECM degradation can be detected, it is possible to accurately identify whether an aneurysm is at risk of rupture. Besides, the potential toxicity of the contrast agent cannot be avoided. The emergence of nanoparticles, which have been used for molecular imaging in targeting cancer cells, shows its essential possibility in aorta diseases (Gao et al., 2012). NP-based molecular imaging technologies have shown impressive efficacy by identifying some critical pathological processes during AAA (Sinha et al., 2014; Ploussi et al., 2015), which offers an exciting opportunity to eliminate the defect in our diagnosis approach beyond size and growth rate. For instance, gold nanoparticles (GNPs), as an ideal radiopaque CT contrast agent, have various advantages, including high density, high atomic number, high X-ray absorption coefficient, and low toxicity (Xi et al., 2012).

Wang et al. (2019) used the AAA model (AngII-induced (LDLr^–/–^) mice fed with a high-fat diet to test their GNPs conjugated to elastin-antibodies (EL-GNP). They demonstrated that EL-GNPs could be successfully targeted to degraded elastin in the diseased aorta, and the accumulation could indicate the level of injury, which is better than the extent of expansion assessed by imaging measurements. This novel targeting technique can predict the level of elastin injury and the possibility of rupture with morphological information, which is superior to imaging modality assessment (Figure 1D). However, the limitation of EL-GNP was still evident that aneurysm couldn’t rupture spontaneously, so all detection was performed *ex vivo*.

Imaging tracers that localize aortic aneurysm-related inflammatory molecules, including 18F-fluorodeoxyglucose (FDG) and ultra-small superparamagnetic iron oxide particles (USPIO), have been deployed in clinical applications. A newly designed FDG-NP, which is to target macrophages, have been tested in AngII-induced AAA with micro-PET/CT imaging (Nahrendorf et al., 2011). They discovered that the amount of NP uptake by the aneurysm had significant predictive value and that low absorption was associated with little expansion over the subsequent 3 weeks, while high NP uptake was related to substantial expansion (Nahrendorf et al., 2011).

Richards et al. (2011) assessed asymptomatic AAAs using the uptake of USPIO NPs to confirm whether inflammation correlated with AAA diameter of 4–6.6 cm. The three USPIO uptake modalities (peripheral-aneurysm uptake only, diffuse patchy uptake within the intra-aneurysm thrombus, and discrete focal uptake distinct from the peripheral-aneurysm region) can be used to predict expansion rates, that patients with significant wall uptake have a three-fold higher annual growth rate than other patients (Richards et al., 2011).

Extracellular matrix is also an attractive target for imaging because the turnover of collagen, an essential component of the ECM, plays a significant role in AA development (Hellenthal et al., 2009). Klink et al. (2011) utilized NPs to functionalize CNA-35, a collagen-specific protein, in an AngII-induced AAA mouse model with TGF-β neutralization (Wang et al., 2010). Intravenous infusion of CNA-35 NPs enhanced the signal in the aneurysmal wall compared to non-specific NPs, and the CNA-35 NPs were shown histologically to colocalize with type I collagen. The higher uptake of CNA-35 NPs is consistent with stable aneurysms which have higher collagen uptake and ruptured aneurysms that have lower uptake collagen content (Klink et al., 2011).

Besides, αvβ3 integrin and vascular endothelial growth factor (VEGF) receptor are both upregulated on neoangiogenic vascular endothelial cells and on inflammatory macrophages. Kitagawa et al. (2012) designed NPs with human ferritin nanocages and Arg-Gly-Asp peptide (RGD) to target integrins, and then image in models (Apo E^–/–^ AngII-induced mice). Using *in situ* and *in vitro* fluorescence imaging followed by NPs with the fluorescent dye Cy5.5, they demonstrated that RGD showed increased uptake of targeted versus compared to non-targeted NPs; by histology, they showed that targeted NPs coincide with macrophage and neovascularization localization (Kitagawa et al., 2012). Tedesco et al. (2009) used similar NPs targeting VEGF in the same model and provided the same tendency. Furthermore, the signal intensity of the aneurysmal segments increased in a diameter-dependent way.

More recently, Yao et al. (2020) evaluated a novel MRI-activatable nanoprobe to label MMPs, which was based on a hydrophilic polyethylene glycol coating immobilized on the outer surface of nuclear/shell iron/iron oxide nanoparticles, coupled with an MMP peptide substrate to enhance their targeting. Probes were injected for the Ang II-induced AAAs mouse model, and MRI detection of aneurysms revealed relatively low contrast noise. Histological examination showed the presence of MMPs and iron oxides in areas with weak MR signals. Nanoprobe-based MRI allows non-invasive detection of MMP activity in the wall of the AAA to predict its risk of rupture.

### Nanoparticles Dynamics

After being introduced in the circulation, the dynamics of NPs vary in the organism. The distribution of iron oxide attached particles in rats is related to the positive and negative charge they carry, and we observe that nanoparticles have the highest accumulation in the liver and spleen when the surface potential is negative, while they have the highest concentration in the lungs when the surface charge is positive. This is due to the magnetism of the carrier (iron oxide) which determines its organ distribution in the body (Sharma et al., 2018). The distribution of GNPs in the body is closely related to the size of the particles, with 10-nm particles widely found in the blood, liver, spleen, kidney, testes, thymus, heart, lung, and brain, whereas for sizes larger than 10 nm they are found only in the blood, liver, and spleen (De Jong et al., 2008).

Understanding the dynamics of nanoparticles in the body is also aimed at reducing the problems they pose. Despite the numerous advantages of NPs, their toxicity is still an important issue to be addressed. A review by Katja et al. explored the mechanism of particle uptake by cells and the factors that influence uptake including particle size, shape, surface charge, surface functional groups, and hydrophilicity. Overall, non-phagocytic cells had the highest uptake of NPs around 50 nm, while for phagocytic cells, the results were inconclusive. Increasing the charge also increased the uptake by the cells (Kettler et al., 2014).

At the same time, understanding how NPs are excreted from the body is necessary for clinical applications. Many of the NPs formulations promising for *in vivo* medical applications are large (>5.5 nm) and non-biodegradable, so they cannot be eliminated by the kidneys. The liver pathway is a new direction of current investigations (Poon et al., 2019).

## Discussion

Nano-biomaterials as technological drivers of innovation have opened a new avenue in the treatment of aortic diseases since they have great potential in improving and modernizing therapy and imaging. The nanosystem of delivery drugs is a promising breakthrough to decrease high risk and high mortality. However, despite its beneficial potentials, there are still problems that need to be solved.

Currently, most of the efficacy results are obtained in animal models, and few clinical trials have been conducted. Although animal models can somewhat reconstruct the course of human aortic disease, there are still inevitable differences and shortcomings. The complex hemodynamic changes in aortic disease, for example, are difficult to achieve in animal models. There is also an inescapable difference between cells of animal and human origin in the face of various stimuli, and pathological processes. iPSCs and organ-on-a-chip are potential experimental platforms in future research (Granata et al., 2017; Gold et al., 2019).

Furthermore, future research should focus on a combination of early diagnosis and treatment. It requires a deeper understanding and a more comprehensive study of the pathological process of aortic diseases. The current selection of targets and cytologic markers is still a single factor. If multiple pathologic factors can be combined, it can increase the positive rate of diagnosis and the effectiveness of treatment.

Meanwhile, researchers should consider how to minimize the toxic effects of the drug, develop efficient delivery approaches, and combine functions without negative impact. It is hoped that such nano-biomaterials would have ranges of applications as an essential tool in the clinic.

## Author Contributions

SZ and KZ prepared the manuscript. JL, HL, and CW proposed the review topic, led the project, and co-wrote/revised the manuscript. All authors contributed to the article and approved the submitted version.

## Conflict of Interest

The authors declare that the research was conducted in the absence of any commercial or financial relationships that could be construed as a potential conflict of interest.
